# Inflammatory Breast Cancer and Warm Antibody Autoimmune Hemolytic Anemia: A Rare Paraneoplastic Syndrome

**DOI:** 10.1177/2324709617740905

**Published:** 2017-11-13

**Authors:** Nene Ugoeke, Chidinma Onweni, Jennifer Treece, Vandana Pai, Sowminya Arikapudi, Evan Kulbacki, Kailash Bajaj

**Affiliations:** 1 East Tennessee State University, Johnson City, TN, USA; 2 Holston Valley Medical Center, Kingsport, TN, USA

**Keywords:** autoimmune hemolytic anemia, inflammatory breast cancer, solid tumor, paraneoplastic syndrome, warm antibody

## Abstract

Autoimmune hemolytic anemia (AIHA) is a disease process that involves the destruction of red blood cells mediated by the humoral immune system. It can be characterized as a cold agglutinin syndrome, paroxysmal cold hemoglobinuria, and warm, mixed type, and drug-induced AIHA. Although a well-established relationship exists between the presence of AIHA and lymphoproliferative malignancy, AIHA rarely presents in association with solid malignancies. An analysis of the limited number of published cases of AIHA in association with solid malignancies performed showed that AIHA may present before the diagnosis of a solid malignancy, concurrently with the presence of a solid malignancy, or even on resolution of a solid malignancy. Few cases of solid cancers associated with AIHA have been reported. AIHA rarely presents as a paraneoplastic syndrome indicating existence of a solid cancer. We report a case of inflammatory breast cancer with AIHA.

## Introduction

The association between autoimmune hemolytic anemia (AIHA) and lymphoproliferative disorders is well-established.^1-3^ However, significantly fewer cases of AIHA have been reported in malignant solid cancers,^4^ with a notably rare occurrence in breast malignancy. To diagnose AIHA in the setting of a solid tumor, a solid tumor must be present and the diagnosis of AIHA must be made using both direct and indirect Coombs tests and presence of anti-erythrocyte antibodies. In a 2016 Bulgarian study that focused on assessing the incidence of autoimmune disorders and solid tumors, out of the 1083 patients with solid tumors that were studied, only 1.29% of the solid tumors were associated with autoimmune paraneoplastic syndromes. Of the solid tumors that also had autoimmune paraneoplastic syndromes, 14% were associated with AIHA, and these were related to either a solid tumor of the prostate or of the ovary with none associated with a solid breast malignancy. AIHA is also found in renal cell carcinoma and Kaposi sarcoma. Idiopathic autoimmune disorders differ from autoimmune paraneoplastic phenomenon by the timing of the development of the disorder as autoimmune paraneoplastic phenomena develop either at the same time as the primary tumor or following the development of the tumor.^5^ There are 2 reported cases in the literature that show an associated malignant mesothelioma solid tumor with AIHA.^6^ There are only a few reported cases of malignant solid breast cancer with AIHA.^7,8^ In a study of 160 patients with both erythrocyte autoantibodies and cancer, a *P* value of <.0005 was found for a single patient to have both erythrocyte autoantibodies and cancer, suggesting that the existence of the 2 conditions within one patient is statistically significant and tends to occur more often with metastatic cancers, large tumors, and is indicative of a poor prognosis. A total of 12.5% of the patients with both erythrocyte autoantibodies and cancer had breast cancer as their primary tumor, and 7.5% of these patients had warm autoantibody AIHA and breast cancer, similar to the patient presented in this case report.^9^ The existence of additional cases of AIHA and breast cancer suggests that there is possibly an association between solid breast malignancy and AIHA. Temporal associations observed in some cases suggest a potential role of AIHA as an indicator of disease remission, progression, or recurrence.

We report a rare case of AIHA in inflammatory breast cancer.

## Case Presentation

The patient is a 40-year-old Caucasian female who presented with a 2-week history of nonproductive cough, generalized weakness, dyspnea, and subjective fever. Examination revealed a large mass in the left breast with near-complete replacement of the breast tissue. There was associated erythema, induration, and skin ulceration with bilateral palpable axillary lymphadenopathy. Complete blood count with differential revealed the following: white blood cell count 7500/µL, hemoglobin 4.0 g/dL, hematocrit 13.4%, platelets 119 000/µL, 43% segmented neutrophils, 22% lymphocytes, 8% monocytes, 2% basophils, and 23% metamyelocytes. Additional workup revealed a haptoglobin level <10 mg/dL, a lactate dehydrogenase level of 1282 U/L, a reticulocyte count >19%, and an immunoglobulin G (IgG) positive direct Coombs test, suggesting AIHA. Based on these findings, the patient received multiple transfusions of packed red blood cells in addition to methylprednisolone for her hemolytic anemia.

**Figure 1.**
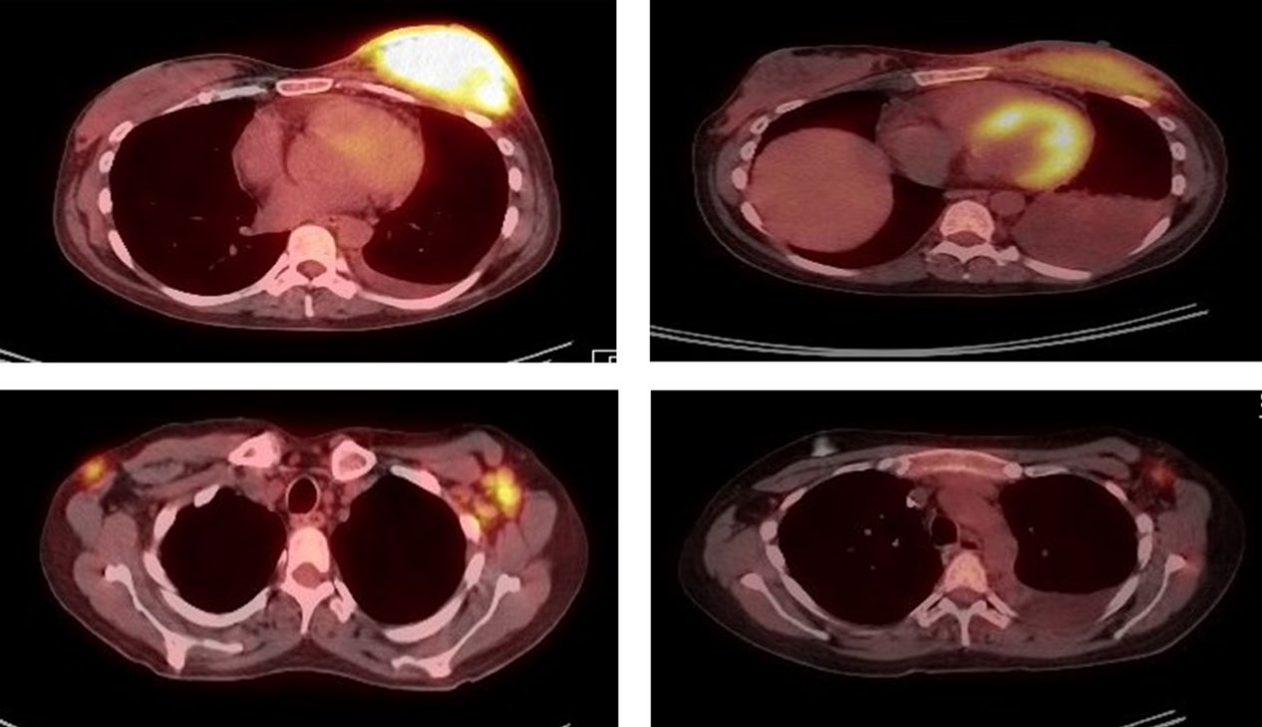
PET-CT at diagnosis and following 6 cycles of chemotherapy. Left column: PET-CT at diagnosis prior to chemotherapy. Right column: PET-CT following 6 cycles of chemotherapy.

Chest computed tomography (CT) scan showed left breast enlargement, left pleural effusion, and diffuse lymphadenopathy. Breast biopsy showed invasive carcinoma with involvement of the overlying skin and lymphatic system. Immunohistochemical analysis showed that the tumor was estrogen receptor positive and progesterone receptor negative. Fluorescence in situ hybridization (FISH) evaluation was positive for HER2 gene expression. Positron-emission tomography (PET) scan showed hepatosplenomegaly, a left breast mass measuring 10 cm × 9 cm × 5 cm, bilateral axillary lymphadenopathy, and a hypermetabolic 6.6 cm diameter pelvic mass. The patient was diagnosed with stage IV invasive ductal carcinoma. She received palliative chemotherapy consisting of docetaxel, transtuzumab, and pertuzumab. Repeat PET-CT scan following 6 cycles of chemotherapy showed improvement in the breast mass and lymphadenopathy, as seen in Figure 1.

However, there was progression of metastatic breast cancer involving the sternum and parasternal soft tissues; multiple cervical thoracic, portacaval, and pelvic nodal metastases; and extensive left side pleural metastases. The abdominal mass pathological result reported the abdominal mass diagnosis as leiomyoma. It lacked atypia and had extremely low mitotic rate, which is as compatible with a benign process. Notably, with response to chemotherapy, there was a decrease in hemolytic activity, and the patient was weaned off steroids. There was a subsequent increase in hemolytic activity after cycle 6 of chemotherapy, however, possibly correlating with progression of metastatic breast cancer.

## Investigations

Evaluation of inflammatory breast cancer and warm antibody hemolytic anemia (AIHA) includes collection of images, laboratory studies of blood samples, and histopathological sampling of tissue. Dynamic contrast-enhanced magnetic resonance imaging provides the ability to assess multicentric and/or multifocal disease along with skin involvement, whereas mammography has shown limited sensitivity. PET is being used for staging and for detecting early metastatic disease.^10^ For our patient, we diagnosed using CT scan, PET scan, immunohistochemical analysis, and FISH evaluation. Blood work includes complete blood count, reticulocyte count, lactate dehydrogenase, haptoglobin, and Coombs test. Breast tissue biopsy provides the definitive diagnosis.

## Treatment

Treatment of the AIHA depends on the characteristics of the concurrent solid tumor. For this patient’s breast cancer, which was estrogen receptor positive, progesterone receptor negative, and positive for HER2 gene expression, docetaxel, transtuzumab, and pertuzumab were initiated. AIHA improved with chemotherapy treatment of the breast solid tumor, suggesting that the solid breast tumor was the underlying etiology of the AIHA. Recurrence of the AIHA following 6 cycles of chemotherapy in a patient with progression of metastatic breast cancer suggests that the recurrent AIHA may be secondary to the progression of the metastatic breast cancer that has become unresponsive to the chemotherapy.

## Discussion

This is a rare case of infiltrative inflammatory breast cancer with a solid mass presenting with concurrent warm antibody AIHA. Mechanisms underlying associations between solid tumors and AIHA remain unclear. However, correlation of hemolytic activity with disease progression or recurrence might be potentially useful in monitoring disease. Further study of patients with breast cancer and AIHA will provide more information on underlying etiology mechanisms.

Inflammatory breast cancer has a very poor prognosis. Molecular subtyping has limited predicted and prognostic value. Triple-negative inflammatory breast cancer is strongly associated with a poor prognosis compared with other subtypes of inflammatory breast cancer, which include hormonal receptor and HER2-defined subtypes. Currently available treatments are unlikely to improve the outcomes. A HER2-positive status does not necessarily indicate a good prognosis as targeted therapies are not effective in terms of improving outcomes.^11^

Inflammatory breast cancer has been reported as a very aggressive form of breast cancers. This was seen in our patient despite prompt treatment and initial partial response to chemotherapy; the metastases at other sites continue to progress despite the comprehensive multidisciplinary approach to the management of this disease, so the prognosis remains dismal.^12^

Chemotherapy that is used to treat the solid tumor will cause the secondary AIHA to respond with a decrease in hemolytic activity as well if AIHA is due to the solid tumor. A response in the AIHA following chemotherapy initiation suggests that the solid tumor is also responding to the chemotherapy. The AIHA initially responded following initiation of chemotherapy, suggesting initial response of the breast tumor to the chemotherapy. AIHA returned with an increase in hemolytic activity as the breast cancer progressed and metastasized further.

## Conclusion

If treatment of the solid tumor with chemotherapy resulted in improvement of the AIHA, then it is highly likely that the AIHA was secondary to the solid tumor. The AIHA level can be used to determine the responsiveness of the tumor to the chemotherapy.^13^ AIHA is a rare paraneoplastic syndrome that has been reported in very few cases to be associated with ovarian cancer, lung, stomach, and breast cancers. Some of the cases reported occurred after or during treatment with carboplatin. Our patient was not on any treatment prior to her presentation. Her presenting symptom was symptomatic anemia. Workup for the anemia confirmed hemolytic anemia. Biomedical research is needed to understand the pathophysiology of AIHA in inflammatory breast cancer and its potential role as a tool in the monitoring of response of inflammatory breast cancer to chemotherapy treatment.
